# “So we brought these players together”: a qualitative study of educators’ experiences to analyze the challenges of creating an e-learning program for neuropalliative care

**DOI:** 10.1186/s12909-024-05437-8

**Published:** 2024-05-10

**Authors:** Julia Bu, Susan DeSanto-Madeya, Mara Lugassy, Jessica Besbris, Sarah Bublitz, Neha M. Kramer, Roop Gursahani, Winnie Lau, Estella Kim, John Y. Rhee, Piret Paal

**Affiliations:** 1 University of California San Diego, San Diego, CA USA; 2 College of nursing, University of Rhode Island, Rhode Island, USA; 3 Westchester Medical Center, Valhalla, NY USA; 4 Cedars-Sinai Medical Center, Los Angeles, CA USA; 5 Institute of Palliative Care, Paracelsus Medical University, Salzburg, Austria; 6 Rush University Medical Center, Chicago, IL USA; 7 P. D. Hinduja National Hospital, Mumbai, India; 8 University of North Carolina, Chapel Hill, NC USA; 9 University of California, Berkeley, Berkeley, CA USA; 10 Dana Farber Cancer Institute, Harvard Medical School, Boston, MA USA; 11 Department of Ethnology, Institute of Cultural Studies, University of Tartu, Tartu, Estonia; 12 Institute of Palliative Care, Paracelsus Medical University, Salzburg, Austria

**Keywords:** Neuropalliative education e-learning

## Abstract

**Background:**

In recent years, the subspecialty of neuropalliative care has emerged with the goal of improving the quality of life of patients suffering from neurological disease, though gaps remain in neuropalliative care education and training. E-learning has been described as a way to deliver interactive and facilitated lower-cost learning to address global gaps in medical care. We describe here the development of a novel, international, hybrid, and asynchronous curriculum with both self-paced modules and class-based lectures on neuropalliative care topics designed for the neurologist interested in palliative care, the palliative care physician interested in caring for neurological patients, and any other physician or advanced care providers interested in neuropalliative care.

**Methods:**

The course consisted of 12 modules, one per every four weeks, beginning July 2022. Each module is based on a case and relevant topics. Course content was divided into three streams (Neurology Basics, Palliative Care Basics, and Neuropalliative Care Essentials) of which two were optional and one was mandatory, and consisted of classroom sessions, webinars, and an in-person skills session. Evaluation of learners consisted of multiple choice questions and written assignments for each module. Evaluation of the course was based on semi-structured qualitative interviews conducted with both educator and learner, the latter of which will be published separately. Audio files were transcribed and underwent thematic analysis. For the discussion of the results, Khan’s e-learning framework was used.

**Results:**

Ten of the 12 participating educators were interviewed. Of the educators, three identified as mid-career and seven as senior faculty, ranging from six to 33 years of experience. Nine of ten reported an academic affiliation and all reported association with a teaching hospital. Themes identified from the educators’ evaluations were: bridging the global gap, getting everybody on board, defining the educational scope, investing extensive hours of voluntary time and resources, benefiting within and beyond the curriculum, understanding the learner’s experience, creating a community of shared learning, adapting future teaching and learning strategies, and envisioning long term sustainability.

**Conclusions:**

The first year of a novel, international, hybrid, and asynchronous neuropalliative care curriculum has been completed, and its educators have described both successes and avenues for improvement. Further research is planned to assess this curriculum from the learner perspective.

**Supplementary Information:**

The online version contains supplementary material available at 10.1186/s12909-024-05437-8.

## Background

Though international humanitarian organizations consider palliative care an international public health priority [1], there is a large unmet global need for palliative care that is projected to grow wider in the coming years [2]. While models of palliative care originally focused on patients with cancer, the subspecialty of neuropalliative care developed to meet the unique needs of patients and caregivers suffering from neurological disease [3], as these patients uniquely face high symptom burden, functional decline, significant care giver needs, prognostic uncertainty, and complex decision making throughout the disease course [4]. Evidence has shown that implementation of palliative care improves patient and caregiver outcomes [3], as neuropalliative care strives to alleviate common sources of patient suffering by focusing on clear and compassionate communication, physical and spiritual symptoms support, caregiver support, and advance care planning [3].

There is, however, no uniformly agreed-upon optimal international model of palliative care for patients living with neurological disease [5]. A roadmap was published in 2018 as a framework for palliative care development in resource limited countries [6]. Possible proposed models include integrative (a neurologist with a specialist palliative care provider), disease specific interdisciplinary clinics, community-based palliative care services, and/or a specialized neuropalliative care provider [7], though as of 2020, there were only 63 neurologists who had active board certification in Hospice and Palliative Medicine (N. Kramer, personal communication, March 22, 2021).

Often cited barriers for integrating neuropalliative care into practice include education gaps of not only clinicians, including neurologists, palliative care providers, and primary care providers, but also patients and communities [3]. An ideal skillset for a neuropalliative clinician includes effective communication skills, navigating complex patient centered decision-making, managing end-of-life symptoms, and providing treatment options and anticipatory guidance [5]. Various curricula have been developed to disseminate core palliative skills, with online education being particularly helpful in providing lower-cost education, addressing global gaps in available resources that often primarily affect low-to-middle income countries.

E-Learning has been described as an innovative approach for delivering a learner-centered, interactive, and facilitated learning environment to anyone, anyplace, anytime, by utilizing the attributes and resources of various digital technologies along with other forms of learning materials suited for open and distributed learning environments. However, success in an e-learning system involves a systematic process of planning, designing, evaluating, and implementing online learning environments where learning is actively fostered and supported [8]. These initiatives range from smartphone application-based or online curricula [9] to Youtube videos [10]. In US-based online curricula in both primary palliative care [11] and neurological subspecialty [12], participants self-reported positive increase in knowledge and competence. Earlier studies on e-learning curriculum toolkit development in neuropalliative care focused on specific diseases [13]. Further creative approaches are needed to meet the need for palliative care expertise globally, and no prior online curricula has focused on neuropalliative education specifically.

We describe here the development of a novel, international, hybrid, and asynchronous curriculum with both self-paced modules and class-based lectures on neuropalliative topics. The course was targeted at physicians and advanced practice providers: neurologists interested in palliative care, palliative care physicians interested in learning unique aspects of care for patients with neurological disease, and any other clinician or advanced practice providers wishing to learn about the intersection of neurology and palliative care. We discuss the structure of this curriculum and explore the educators’ experiences. Since work on palliative care curricula is chronically underfunded, this paper provides some “lessons learned” to help with the general organization of e-learning.

### The overall objectives of the E-learning program

The overarching objectives for this year-long course were to:

1. Bridge educational gaps by:
  - teaching palliative care basics to neurologists and other non-palliative providers.
  - teaching neurology basics to palliative care providers and other non-neurologists.
2. Provide a comprehensive neuropalliative skill set that will enhance all participants’ clinical approach to people living with serious neurological conditions.
3. Create a repository of multimodal resources (e.g. webinars, readings, interactive sessions) for use during and after the course.
4. Bring together interdisciplinary professionals in neurology, palliative care, and neuropalliative care in a shared environment to foster collaboration and networking across disciplines.

## Methods and curriculum description

The course consisted of 12 modules, one per every four weeks over a twelve month period, beginning July 2022. Course participants from a variety of professional backgrounds were invited to participate, including neurologists, neurosurgeons, palliative care providers, geriatricians, physiatrists, internists, intensivists, pediatricians, and advanced practice providers. The course offering was listed in Indian neurology and palliative care websites and communication groups, as well as the International Neuropalliative Care Society website. Thirty-nine clinicians and one advanced practice provider registered for the course. Course content was divided into three streams: Neurology Basics, Palliative Care Basics, and Neuropalliative Care Essentials. Learners were encouraged to explore the first two streams to fill foundational gaps based on their training background and practice specialty, and all were expected to participate in the neuropalliative stream. Topics and objectives for each stream can be found in Table 1 and supplemental Table 1 respectively. Each module was built around an exemplary case. Traditional, bottom-up neurological education, from basic science through clinical skills and then on to diseases, is unworkable for non-neurologists [14] and likely contributes to “*neurophobia”* [15]. This course used a phenomenological approach to enable learners to fill neuropalliative care knowledge and skill gaps using clinical context and relevance. These strategies included pre-recorded webinars and readings, group and individual assignments, monthly flipped classroom interactive sessions, and summative assessments including multiple choice questions and reflective exercises. The flipped classroom consisted of a case presentation and additional materials to supplement the webinar content, with open discussion from learners and educators. Learners were asked to complete a post-course evaluation, the results and analysis of which will be published separately. Learners were expected to spend an average of 2–3 h per week on course work. In order to pass the course, learners were required to attend 80% of flipped classroom sessions, complete 80% of Neuropalliative Care Essentials webinars, receive an 80% pass rate on multiple choice questions, and submit written assignments for each module. Learners were also expected to attend an 8-hour in-person session focused on developing neuropalliative communication skills, with additional opportunity for networking across disciplines.

**Table 1:** Course Content and Structure

Module			Webinar list	Case
	Neurology	Palliative Care (PC)	Neuropalliative care (NpC) essentials	
1	Neurology localization I	Introduction to PC	Introduction to NpC	59/F with Glioblastoma
	Brain and spinal tumors	Comprehensive needs assessment	Disease trajectories in Neurology	
			Neuro-oncology overview	
2	Neurology localization II	Communication skills in Serious Illness	Pediatric NpC basics	16/M, metastatic osteosarcoma
			Malignant spinal cord compression	
		Pain: assessment and management	Living with severe Spinal cord injury	
3	Parkinson disease and related disorders (PDRD)	Recognition and management of psychological issues in PC	Serious illness communication in Neurology	45/M, Idiopathic Parkinson disease
			PDRD: Psychosocial needs and non-motor symptoms Part I	
			PDRD: Non-motor symptoms Part II	
4		GI symptoms in PC	Neuropsychological manifestations in PDRD	78/F, Advanced idiopathic PD
			PC needs in advanced PDRD	
			Management of end-stage PDRD	
5	Unconscious patient and Serious Acute Brain Injury (SABI)	Principles of Prognostication	Clinical application of medical ethics in neurological disease	21/F, Severe traumatic brain injury
			SABI: Clinical prognostication and outcomes	
			Prognostic uncertainty: management and communication	
6	Stroke basics	Limiting life sustaining treatment	Chronic disorders of consciousness (C-DoC): classification	45/M, Brainstem stroke
			C-DoC: legal and ethical issues	
			Goals of Care in life limiting neurological illness	
7	Delirium and Dementia	Advance care planning	Overview of dementia through the PC lens	73/F, Mild to moderate dementia
			PC in early onset and non-Alzheimer dementia	
			ACP in Mild dementia	
8		Opioid basics	Behavioral and Neuropsychologic symptoms in Dementia	73/M, Advanced Lewy body dementia
			End of Life care in advanced dementia	
			Supporting care partners in advanced dementia	
9	Approach to lower motor neuron disorders	Respiratory symptom management	Symptom management: Motor neuron disease (MND), Part I	53/M, MND
			Symptom management: MND, Part II	
		Principles of End-of-Life care	Decision points and End-of-life care for MND	
10	Childhood Chronic neurological Illnesses: transitions to adulthood & PC	Spiritual dimensions of care	Management of Dysarthria & Dysphagia in neurological disease	19/M, Duchenne muscular dystrophy
		Requests for Hastened/Assisted Death:	Rehabilitation in progressive neuromuscular disease	
			Shared Decision Making in Paediatric Patients	
11	Infectious and inflammatory CNS disease	Hospice: transitions and organization	Neuropathic pain: management principles	21/F with multi-drug resistant tubercular meningitis
			Bladder & Urinary Catheter management in Neuro disease	
		Pressure ulcers	Delirium in neurological illness	
12	Common neurological symptoms	Palliative Sedation	Clinician burnout, resilience, self-care	45/M, Leuko-dystrophy
		Grief & Bereavement Support	Seizure management at the end of life	
			Planning and setting up a Multidisciplinary team	

## Evaluation of the E-learning program from the educators’ perspective

To evaluate the e-learning program, a two-stage evaluation strategy based on semi-structured qualitative interviews was proposed to explore the experiences of educators and learners. In this paper, we report on educator experiences.

A semi-structured interview guide was developed by the research team. The interview guide consisted of four main questions: (1) How was your personal experience in developing this online course? What went well? What were the biggest challenges that had to be overcome? (2) Do you think this online course is a good model for the development of palliative care among healthcare professionals, and why? (3) What parts of the course did you find particularly useful? And where do you think the course could be improved? (4) And finally, what is your personal opinion of this online course? Any wishes for the future? The interviews were conducted in English with a recognition that this was not the native language of all educators.

Twelve experts involved in the curriculum’s development were identified and invited via email to participate in the study. The invitation included a statement, the interview questions, the purpose of data collection, and an informed consent statement. An appointment was then made for the interview. Interviews were conducted by two researchers using online videoconferencing. Introductory interviews were conducted prior to audio and video recording.

### Ethical approval

Interviews were conducted as a component of program evaluation. Participation in the evaluation process was voluntary. Verbal and written consent to record and use the data for research purposes was obtained from all participants before the video recording began.

### Data analysis

The audio files were transcribed verbatim and prepared for thematic analysis using Excel. First, the researchers familiarized themselves with the verbatim transcripts. Second, codes were identified by highlighting units of meaning in the text and labeling them to describe their content. Third, patterns between codes were identified, and codes were grouped together to identify themes. The themes were reviewed by splitting, combining, discarding, or recreating themes to make them more useful and accurate. Finally, the final list of themes was transferred to Miro Board to formulate the final list of themes. The formulation was done by a research group that discussed what was meant by each theme and how it contributed to the understanding of the data.

### Data synthesis

For the discussion of the results, Khan’s e-learning framework with eight dimensions was applied to this work [8]: institutional, management, technological, pedagogical, ethical, interface design, resource support, and evaluation, described further in results section.

## Results

Of 12 educators, 10 agreed to participate in the evaluation. These included six from palliative care and four from neurology/neuropalliative care backgrounds. One was a nonclinical researcher. Four were from the USA, five were from India, and one was from Europe. Of the educators, three identified as mid-career and seven as senior faculty, ranging from 6 to 33 years of experience. Nine of ten reported an academic affiliation and all reported association with a teaching hospital.

The themes identified were as follows: (1) Bridging the global gap; (2) Getting everybody on board; (3) Defining the educational scope; (4) Investing extensive hours of voluntary time and resources; (5) Benefiting within and beyond the curriculum; (6) Understanding the learner’s experience; (7) Creating a community of shared learning; (8) Adapting future teaching and learning strategies; and (9) Envisioning long term sustainability.

### Bridging the global gap

Educators agreed there is a need for education and training in neuropalliative care, though they noted worldwide there are few palliative care courses or the courses are limited to specific health systems or diseases. Educators noted online education and training help to disseminate information to a larger community, as there will be a greater demand for palliative care course, and felt this course needs to continue being offered internationally in the future.

### Getting everybody on board

Educators noted currently there is little research about palliative care education online and found developing and implementing an online course is a navigation of a complex landscape of policies and requirements which may be specific to institutions, healthcare systems, or countries. Additionally, when tailoring a course to meet the goals of an international community, they emphasized local guidelines, cultural norms and systems of medical practice to consider, and a big challenge to get such varied communities on board. For example, one educator noted that in India, views on patient autonomy may be different than the West, which is pertinent for the clinician teaching palliative care to understand.

*“India… is a collectivist society… we as a culture have willingly surrendered a good portion of… our personal autonomy to our families. The golden rule is you treat me like you would like to be treated… But the platinum rule is that you treat me the way I would like to be treated.” (009).*

### Defining the educational scope

Educators noted that neuropalliative care is an innovative specialty-based model of palliative care, and found that though the entire project was ambitious, it was helpful to consider and develop basic knowledge and skills needed to deliver neuropalliative care.

*“I think the stream that brought it all together, these key neuropalliative issues, you know, are really incredibly important to the goal of this course to really focus on specific elements of palliative care for neurological patients.”* (003).

*“So first we talked about who this course is for? Is it for neurologists? Is it for palliative care specialists? Is it appropriate for both? And is it aimed at non-physicians? So we thought about the audience and the medium: should it be in person or virtual? Should it be a combination? These were the first steps.”* (005).

One theme noted by educators was the challenge to focus this course on the specific elements of palliative care for people with neurological problems. The course needed to provide relevant education for both palliative care providers and neurologists, and emphasis was placed on complex decision making. To meet the learners’ expectations and make the course engaging, they noted course content needed to be adapted to answer local questions. For example, changes in content were made to make it appropriate for India:

*“I think if I did my best, I would miss the mark, and I would miss the mark in many ways, because there are nuances in the discussion of advance care planning in India and other countries, there are nuances in policy, there are nuances in the care that is provided and available. So I think having a course that’s really practical and real-world will be helpful for providers. You know, it’s helpful to have people who are on the ground and really know these systems.”* (002).

### Investing extensive hours of voluntary time and resource

“*This is the most extensive course I have ever tried to create!*” (005).

Educators acknowledged the discussion began during the [Covid-19] pandemic and the contemplation period took more time than expected. Coordination of a large planning group was complex and meticulous. It took numerous long phone calls to understand how online learning works and several months to discuss the curriculum content.

“*So we brought these players together for different groups of people and found a common time. I think that was one of the biggest challenges in working together and creating something where everyone could come together. It was difficult, but the result was very positive because everyone was willing to put in the time that was required.*” (007).

Despite being pro-bono work, educators admired how many people volunteered to create this program and its content, bringing various resources together to provide a rich and interactive curriculum.

### Benefiting within and beyond the curriculum

Educators described benefiting beyond the curriculum itself as being a part of the curriculum designers and learning from international colleagues.

*“It’s a journey for me… because this is a first for me. I wasn’t earlier part of any other course module or development techniques.”* (001).

Educators reflected that putting the curricula itself together allowed them to learn not only about neuropalliative care, but about what topics may be important to local practice.

*“I’m 63, that’s not an age at which I’m going to go do a fellowship now… the best way to learn is to teach”.* (006)

### Understanding the learner’s experience

Educators noted understanding the audience is vital; the learners were all busy clinicians, and self-paced learning meant that not everyone came prepared. Educators reflected on this lack of engagement and suggested a more rigorous selection process for learners is needed for future offerings of the course. They believed as the goal of a curriculum is for learners to learn, learner engagement and retention of information is imperative. Educators reflected that learner feedback of the course is vital to course evaluation.

*“Something that the group noticed was that the palliative care folks were much more engaged than the neurologists. So we really wanted to understand why that was and what we could improve for the neurologists to make it more interesting for them and make them feel like they could really benefit from this course.”* (005).

Educators reflected that currently how this course impacted learner clinical practice is unknown. Though available feedback can be used to modify the course, reasons for learner attrition is also difficult to ascertain. Educators felt that for future iterations of the course, soliciting feedback from learners at the beginning and middle of the course may aid in these evaluation gaps.

### Creating a community of shared learning

One theme that was reported by educators was that online learning is about building a community, so conversations between course participants are essential. One aim was to make this course a place for networking.

*“You might want to divide the participants into small groups to do small projects together. That way they get to know a few others and have a chance to share ideas and set a goal. I also think that the monthly sessions at via zoom should be much more interactive so that the course participants have a lot more say. I think that would be a space for growth.”* (004).

Educators reflected that content needs to become more concise to allow for synchronous online activities, regular check-ins about personal practice, and discussions about what was learned. Educators felt that small group assignments improve cross-country collaboration, and allowing time for discussion can foster teaching among learners, as practitioners may know more about the community and its needs than some educators.

### Adapting future teaching and learning strategies

Educators identified that there is room for growth; questions abound for what teaching strategies need to be changed next course offering. Currently, the focus is on webinars. Educators felt the content could be consolidated by having some essential knowledge acquisition through self-directed learning and currently the course is very theoretical. They thought it may benefit from more practical cases specific to the clinician’s area of expertise, such as with inclusion of patient and family voices or with more skill development sessions. The bulk of the curriculum focused on current knowledge.

*“Put them in break-out rooms, you know, and discuss face to face. That will enhance our learning. And then give them cases, break them up into groups of five, and show them how they could do it. Those kinds of discussions would show what they have learned over the last month. So we need to incorporate more of these skill-based sessions.”* (007).

In terms of performance assessment, educators felt the MCQs should be replaced at least in part by practical tasks.

### Envisioning long term sustainability

Educators noted in the first year, the goal was to create all the content and get the pilot course started. For subsequent iterations, educators posed questions to consider including: What happens next time? How can awareness be spread about the course? How can more participants attend the live sessions? A course is only helpful if learners complete the course. Would conferring a degree instead of a certificate improve participation? From the educator side, more comfort with the online curriculum is needed:

*“I have some people who are known as educators, but they had a very hard time transitioning to online content and asynchronous content and building a community. If you’re not used to watching YouTube, you may have a hard time engaging with it.”* (008).

## Discussion

The demand for palliative care for people with neurological disease and their caregivers is rising globally. Palliative care education and curricular development is central to improving access to high quality palliative care. Targeted initiatives to improve healthcare workers competencies in symptom control, communication, advance care planning are needed. Integrating palliative care into public health systems is important for the sustainability of palliative care around the world. The resources for planning and creating educational activities ‘*rest on the shoulders of champions*’ [16]; however the lack of explicit funding for creation and maintenance of such educational activities, such as e-learning programs, is a well-known barrier.

For the e-learning program described here, the educators’ evaluations indicate that the program is consistent with the mission of the International Neuropalliative Care Society (INPCS) to build an international neuropalliative care community. This course not only addresses common topics relevant to caring for patients with serious neurological conditions and their caregivers, but it also highlights the importance of multidisciplinary practice and integrative care and provides enhanced opportunities for interprofessional collaboration and education. All these components have been highlighted as priorities in the field of neuropalliative care [5].

There are some lessons to be learned from the educators’ evaluation of the e-learning program relevant to individuals designing a curriculum in a newly defined field, such as neuropalliative care. Khan’s E-Learning Framework with eight dimensions has been applied to this work: institutional, management, technological, pedagogical, ethical, interface design, resource support, and evaluation. All dimensions work to foster analysis and evaluation of every aspect of the e-learning design process [8]. This evaluation identified four main learning points, which are discussed below.

*Allow spaces to learn from learners.* Khan’s E-Learning Framework supports educators’ experiences that managing people, content, and the e-learning environment is time-consuming and largely a voluntary task that requires getting everyone on board. While curriculum developers may feel compelled to provide as much information as possible, socioeconomically and culturally familiar content may be more relevant to learners. Planning events in which learners can learn from each other through discussion and networking can also increase the amount of actionable information shared. Mindfulness of nurturing learning cultures whilst building global learning platforms cannot be overlooked [17]. A recent study proposed four dimensions that can be factors of convergence or divergence for learners: management of time and learning activities, management of the learning space, management of interpersonal relations, and style of communication [18]. Most importantly, online learning networks foster community growth [19, 20] which benefits any new field. Neuropalliative care is a newly defined field with limited availability of evidence-based information. Available evidence is collected from certain populations, socioeconomic and cultural contexts, and therefore, entails little social and cultural diversity. Getting everyone on board means also adding learners’ voices to the curriculum via enabling spaces for exchange, reflection and obtaining new insights from learners [21].

*Plan your e-learning as if there will not be a second time*. The evaluation for e-learning and assessment of learners’ experiences was of concern, which is closely related to the pedagogical and ethical dimensions of e-learning programs. Educators clearly pointed out the monotony in teaching and assessment strategies, as well as the need for changes related to improving the collective learning experience, incorporating patient and caregiver voices, and providing workshops to improve practical skills. Although educators are well aware of the importance of such improvements, the enormous amount of volunteer work involved raises the question of whether educators would be motivated enough to work a second time, adding to concerns of long-term sustainability of such e-learning programs. At this time, similar to the seeds that grew this current project, the field of neuropalliative care relies on practitioners dedicated to the field to continue providing volunteer time to advance the field’s missions, including engaging new communities nationally and internationally [3]. As communities and professional organizations further realize the importance of neuropalliative care in holistically supporting patients with serious neurological disease, it is our hope that educational initiatives like this one continue to flourish.

*Focus on learner progress, support, and professional gains throughout the process.* The institutional dimension addresses administrative, academic affairs, and learner services issues related to e-learning. Different organizations may have different requirements, which can cause delays and interruptions in the process. In the current project, the different requirements were successfully brought together. The dimension of resource support of e-learning to promote meaningful learning was discussed by educators. As learners invest many hours in participating in e-learning programs, the return on investment must be clear. Successful self-directed learning requires commitment and guidance, and therefore, in order to provide the best care for people with neurological conditions, healthcare providers motivated to improve their knowledge and skills must be provided with sufficient learning time, financial support, and professional development. The E-learning module structure was chosen for this current endeavor to balance the motivation of learners with practical availability of time in a clinician’s work schedule for optional learning, though educators felt that certain key clinical skills of the palliative care toolkit such as communication skills are better practiced by live faculty-led sessions. Further work evaluating the learner’s perspectives is needed to elucidate the ideal balance of asynchronous modules vs. live sessions for neuropalliative skills, though the asynchronous module option, touted by this project’s educators as well as in prior literature [11], appears to remain the most practical way to deliver education to a busy and geographically diverse audience.

In terms of e-learning programs, the first step is to help learners determine their motivation for taking the course through screening. Recently novel approaches to predict learners’ success have been proposed [22, 23]. Secondly, regular hands-on assignments and assessments can help motivate and focus learners. Finally, there is the question of whether a certificate is sufficient motivation or whether the course should lead to a degree that can be used to achieve subspecialization in neuropalliative care. An exploration of learners’ feedback from this educational endeavor will be published separately.

*Be aware of your blind spots.* The interface design, online support, and technology infrastructure were not discussed by educators involved, which suggests a knowledge gap in this area, and confirms the perception that online teaching needs new kinds of educators [24]. User friendliness and questions regarding learning infrastructure are important factors that enhance learners’ experience. Professional companies with better understanding of how e-learning functions should be involved from the beginning. In terms of evaluation, it has been indicated that the capability of monitoring and storing user traffic is useful to understand a learner’s behavior, proficiency, accumulated knowledge and learning curve [25], which can be considered for future iterations of this course.

## Conclusions

Success factors in e-learning implementation effectiveness have been related to technology, learner, instructor, content, and institution support [26]. We describe here the educators’ reflections of designing and implementing an international e-learning program in neuropalliative care, highlighting their perceived successes such as bridging gaps in education, building international communities, themselves learning from experts outside the curriculum, as well as avenues for improvement such as in integrating technology [22] and questioning sustainability. Furthermore, analysis of learner evaluations will provide further insights to help understand how this e-learning program is perceived and its educational benefits from the learners’ perspective.

### Electronic supplementary material

Below is the link to the electronic supplementary material.

Supplementary Material 1

## Data Availability

Data sharing is not applicable as no datasets were generated or analyzed during the current study. Copies of interview transcripts are available upon request from the first author.
