# Supplementary material for: “So we brought these players together”: a qualitative study of educators’ experiences to analyze the challenges of creating an e-learning program for neuropalliative care
Source: BMC Med Educ. 2024 May 10;24:524. doi: 10.1186/s12909-024-05437-8 (PMC11088003; doi:10.1186/s12909-024-05437-8)
Supplement: Supplementary file 1 — Supplementary Material 1 [file 12909_2024_5437_MOESM1_ESM.docx]

**Supplemental Table 1: Webinar Topics and Objectives for each stream**

| **Module** | **Case** | **Neurology Basics Webinar Topics & Objectives** | **Palliative Care Basics Webinar Topics & Objectives** | **Neuropalliative Care Essentials**  **Webinar Topics & Objectives** |
| --- | --- | --- | --- | --- |
| 1 | 59/F, R temporal malignant glioma R Temporal | **Neurology Localization Basics Part I**  1. Recognize the importance of localization in neurological diagnosis using the neuraxis chart  2. Describe the localizing value of common symptoms and the time course of major pathologies  3. Explain steps of performing a basic neurological exam (cranial) including speech/language and cranial nerves | **Introduction to Palliative Care**  1.Analyze cardinal concepts underlying the philosophy of Palliative Care based on WHO, IAHPC  2. Evaluate the domains of PC provision as they apply to practice  3. Recommend models of PC delivery suitable for practice | **Introduction to Neuropalliative**  1. Examine the origins of the field of neuropalliative care and review its utility in addressing the needs of patients with neurological illnesses  2. Identify commonly used palliative needs assessment tools for neurological disorders  3. Compare different models for delivery of neuropalliative care  4. Evaluate the challenges in integration of palliative care for different neurological conditions globally |
|  |  | **Brain and Spinal Tumors**  1. Classify brain tumors  2. Recognize broad categories of paraneoplastic syndromes and their management  3. Distinguish extra-dural, intradural and intramedullary myelopathy syndromes | **Comprehensive needs assessments and management**  1. Assess two models of palliative care needs assessment tools (POS, ESAS-r)  2. Explore the values involved in person-centered care.  3. Develop a comprehensive assessment and care plan using Interdisciplinary resources for disease management, physical and emotional symptom management, communication, shared decision-making, practical and social issues, advance care planning | **Disease Trajectories in Neurology**  1. Compare and contrast the trajectories of acute and chronically progressive neurological disease, and with other conditions such as cancer or heart failure.  2. Identify palliative care needs of patients at different points through the trajectory  3. Recognize the importance and inherent challenges of identifying triggers for palliative care referral, given variability in neurological disease trajectories |
|  |  |  |  | **Neuro-Oncology overview**  1. Evaluate the palliative care needs of neuro-oncology patients and their care partners  2.Examine the unique considerations with respect to prognosis, end-of-life, and advance care planning in this patient population  3. Review how to manage the most common symptoms in neuro-oncology patients, especially at the end of life |
| 2 | 16/M, Recurrent osteosarcoma with bony metastases | **Neurology Localisation Basics Part II (Spinal cord and peripheral)**  1. Explain steps of performing a basic neurological exam (spinal, LMN) including motor, reflex, sensory and gait testing  2. Distinguish the applications of various investigations: CT, MRI, EEG, EMG  3. Describe how to put it all together using case vignettes | **Communication Skills and their application in serious illness Part I**  1. Review the concepts of “Goals of Care. and shared decision making  2. Identify key practices for success in discussing goals of care.  3. Apply the REMAP framework to guide goals of care conversations  4. Manage family collusion | **Malignant spinal cord compression**  1. Review the presentation and diagnosis of malignant spinal cord compression  2. Review the approach to management of malignant spinal cord compression, including dexamethasone, radiotherapy and surgery |
|  |  |  | **Assessment and management of pain**  1. Identify types of pain and demonstrate the assessment of a patient in pain using physiologic, etiologic and evaluative approaches.  2.Describe total pain using the biopsychosocial model  3. Demonstrate Management of Pain pharmacological, non-pharmacological and interventional | **Living with severe spinal cord injury**  1. Develop an understanding of the major chapters of life following spinal cord injury, including early rehabilitation, community reintegration, and long-term medical issues/health maintenance/ economic/societal issues  2. Review common physical symptoms after SCI and management strategies including spasms, spasticity, neurogenic bowel, etc. |
|  |  |  |  | **Pediatric Neurology Basics**  1. Define pediatric palliative care and develop a broader context for the practice.  2. Understand the types of conditions and demographics of children receiving pediatric palliative care and highlight the need for expertise in pediatric neuropalliative care.  3. Acknowledge the uniqueness of pediatric palliative care for children with progressive, but non-malignant neurological disease.  4. Understand the approach to management of neurological symptoms in pediatric palliative care. |
|  |  |  |  | **Shared decision-making in pediatric patients**  1.Define and develop an approach to shared- decision-making in pediatric patients  2. Understand the challenges and adaptations of shared decision making in pediatrics  3. Develop strategies to address care partner requests to withhold information from patients |
| 3 | 36/M, Young Onset Parkinson’s Disease | **Parkinsonism**  1. Classify movement disorders 2. Recognize idiopathic Parkinsonism stages and explain management of early and advanced PD  3. Distinguish individual Park plus syndromes | **Recognition and Management of Psychological issues in Neurology and Palliative Care**  1. Understand the illness experience in chronic illnesses – the bio-psycho-socio-spiritual model  2. Explore the tools for identifying psychological issues in palliative care (Distress Thermometer, GAD-7, PHQ-9)  3. Understand basic pharmacological and non-pharmacological management of anxiety, depression, demoralization and adjustment disorders | **Serious illness communication in Neurology**  1. Implement SPIKES and other frameworks for delivering difficult news  2. Recognize the cognitive trap and respond to emotion using NURSE statements  3. Demonstrate approaches for formulating and communicating prognostic information |
|  |  |  | **Communication Skills and their application in serious illness – Part I**  1.Reflect on the impact of skilled and compassionate communication on patient care and provider satisfaction.  2. Implement the SPIKES framework for delivering “difficult news”  3. Use NURSE statements to respond to emotion | **Psychosocial needs and non-motor symptoms Part I**  1. Recognize common existential concerns in persons newly diagnosed with Parkinson’s disease.  2. Differentiate the psychosocial needs of young-onset Parkinson’s disease patients from the needs of adults who are older at the time of diagnosis.  3. Diagnose and manage common non-motor symptoms that impact quality of life in patients with Parkinson’s disease and related disorders, including sialorrhea, sleep disorders, fatigue, depression/anxiety, restless leg syndrome. |
|  |  |  |  | **Non-motor symptoms Part II**  1. Describe dietary, behavioral and pharmacological approaches to treating constipation and gastroparesis  2. Outline an approach to the management of orthostatic hypotension, incorporating non-pharmacological and pharmacological approaches  3. Describe symptoms of sexual dysfunction in men with Parkinson’s disease, and common treatments |
| 4 | 73/F, Hypertension, Diabetes Mellitus, Coronary artery disease, Parkinson’s Disease Stage 4, Deep Brain Stimulator at age 68 | **The Unconscious Patient and Serious Acute Brain Injury (SABI) - Part 1**  1. Distinguish structural versus non-structural mechanisms for coma;  2. Recognize clinical features of herniation and their importance  3. Compare GCS and 4-score scale  4. Identify major pathologies on CT scan | **GI Symptoms: Nausea, Vomiting, Constipation**  1. Describe the pathophysiology of nausea, omitting, and constipation  2. Demonstrate the pharmacological and nonpharmacological approach nausea & vomiting.  3. Demonstrate the pharmacological and nonpharmacological approach to constipation. | **Neuropsychiatric manifestations in Parkinson’s Disease and Related Disorders (PDRD)**  1. Develop a plan to treat visual hallucinations, psychosis, and impulse control disorder, including behavioral interventions and management of dopaminergic and non-dopaminergic medications  2. Discuss the role of and process of deprescribing in advanced PDRD. |
|  |  |  | **Opioid basics**  1. Explain opioid Pharmacology  2. Demonstrate appropriate patient selection, opioid prescription and opioid conversion & rotation.  3. Identify and handle opioid toxicity and describe risk mitigation strategies | **PC needs in advanced PDRD**  1. Explore evolution of motor and non-motor symptom burden over the course of Parkinson’s disease and related disorders.  2. Describe types of pain in Parkinson’s disease and an approach to treatment.  3. Identify factors that contribute to existential distress and care partner burden in advanced Parkinson’s disease. |
|  |  |  |  | **Management of end-stage PDRD**  1. Identify prognostic indicators in PDRD  2. Identify “milestone” events that should prompt a serious discussion and evaluation of goals of care.  3.Describe typical locations and mechanisms to provide end-of-life care to patients with Parkinson’s disease.  4.Name unique aspects of hospice care for patients with PDRD |
| 5 | 16/F, severe traumatic brain injury | **The Unconscious Patient and Serious Acute Brain Injury (SABI) - Part II**  1. Recognize the consequences of traumatic brain injury and specify approaches to management  2. List steps involved in making a diagnosis of brain death  3. Identify the chronic disorders of consciousness | **Principles of Prognostication**  1. Discuss palliative prognostication using prognostic tools and clinical prediction  2. Defend the bioethical justification for prognostication  3. Suggest approaches for formulating and communicating prognostic information  4. Utilize prognostication to guide clinical decision-making | **Clinical application of medical ethics in neurological disease**  1. Review basic principles medical ethics and their application to neurological disease  2. Define selective paternalism as it applies to care of the severely and irreversibly brain-injured person  3. Differentiate "best-interests" decision-making versus substituted judgment, for incapacitated patients |
|  |  |  | **Withholding vs withdrawing life sustaining treatment**  1) Define withholding and withdrawing LST and how they differ from euthanasia and /assisted death 2) Evaluate the 4-box model of medical ethics in the context of medical futility  3) Define the terms AND/DNAR, palliative extubation, withdrawal of CANH and POLST/MOLST | **Severe Acute Brain Injury (SABI): clinical prognostication and outcomes**  1.Identify the classic illness trajectory of SABI  2. Define the “self-fulfilling prophecy” as it applies to SABI outcomes  3. Identify the limitations of prognostic indicators/scores in the setting of SABI. |
|  |  |  |  | **Prognostic uncertainty: management and communication**  1. Acknowledge the importance of prognostic disclosure in the setting of uncertainty.  2. Illustrate techniques and frameworks to communicate prognostic uncertainty, including avoidance of ambiguity/hedge language and best case/worst case framework  3. Demonstrate techniques to manage prognostic uncertainty including the use of time limited trials |
|  |  |  |  | **Severe Acute Brain Injury (SABI), brain death and palliative extubation**  1.Differentiate SABI vs brain death  2. Explore unique communication techniques when discussing brain death with surrogates, and how these differ from conversations about other forms of SABI  3. Identify common symptoms/manifestations to be managed in compassionate extubation of a severely neurologically injured patient, including identification of signs of imminent death. |
| 6 | 45/M, Brainstem and diencephalic stroke | **Stroke**  1. Distinguish various stroke subtypes and itemize severity using the NIHSS  2. Recount basic steps of management of ischemic stroke and hemorrhagic strokes, including subarachnoid hemorrhage | **The last chapter: end of life (EOL) Care**  1. Recognizing EoL Trajectories, terminal illness and imminent death.  2. Review the 6-step approach for End-of-Life care.  3. Identify and describe in brief the care of adults in the last days of life, including management of distressing symptoms and anticipatory prescribing | **Chronic Disorders of Consciousness (C-DOC): Classification**  1: Define the three major categories of C-DOC  2: Define the criteria for emergence from a minimally conscious state (MCS)  3: Describe at least two common mimics of a C-DOC |
|  |  |  |  | **Chronic Disorders of Consciousness (C-DOC): Legal and ethical issues**  1: Define “therapeutic nihilism” as it pertains to patients with C-DOC  2: Describe at least one seminal legal case surrounding the rights of continuation or discontinuation of life support for patients with C-DOC  3: Name at least one recent advancement in evaluation of awareness in patients with C-DOC |
|  |  |  |  | Goals of Care in life-limiting neurological illness  1. Describe the approach to goals of care discussions for patients with life-limiting neurological illnesses  2. Evaluate opportunities for proactive goals of care discussions based on expected clinical trajectories and clinical triggers  3. Identify patient, care partner, clinician, and systemic barriers to goals of care discussions |
| 7 | 68/F, Mild cognitive impairment, confirmed Alzheimer's dementia | **Dementia**  1. Distinguish delirium and dementia with essentials of testing for both states  3. Distinguish Alzheimer and non-Alzheimer degenerative dementias and HANDS  4. Stage dementia progression | **Advance Care Planning**  1. Define Advance Care Planning, its essential elements and the process of framing Advance Medical Directives  2. Explain the evolution of patient rights at the end of life and the systems that make these possible across the world.  3. Define the different types of decision-makers including guardians, DPOA, and surrogate decision makers  4. Discuss the challenges to Advance care Planning | **An Overview of Dementia Care Through the Lens of Palliative Care**  1. Consider strategies for sharing a diagnosis of pre-clinical or early dementia  2. Identify stages of dementia, prognostic indicators, and the limitations of existing prognostic tools  3. Identify techniques for providing anticipatory guidance and assistance with transitions between dementia stages. |
|  |  |  |  | **Early Onset *(and Non-Alzheimer’s*) Dementia**  1. Review unique needs of patients with early onset Alzheimer’s disease and other atypical forms of dementia  2. Compare and contrast approaches to the palliative management of early onset dementia compared to late onse.  2. Review the current pharmacologic approaches to managing cognitive impairment in Alzheimer’s disease and related dementias, including deprescribing |
|  |  |  |  | **Advance Care Planning in Mild Cognitive Impairment and Mild Dementia**  1. Define and develop an approach to assessing decision making capacity  2. Apply an understanding of advance care planning in the context of mild cognitive impairment and mild stage dementia  3. Review approaches for initiating advance care planning in early dementia  4. Provide an overview of communication skills and methods to improve the quality of goals of care discussions with patients living with dementia and their care partner |
| 8 | 73/M with Dementia with Lewy Bodies, transitioning from moderate to advanced stage dementia | None | **Pressure ulcers**  1. Evaluate risks for development of pressure ulcers in a patient with neurological illness  2. Describe pathophysiology and classify pressure ulcers  3. Demonstrate strategies for prevention and management of pressure ulcers in a bed-ridden patient. | **Behavioral & Neuropsychiatric Symptoms in Dementia**  1. Provide an overview of the phenomenology of behavioral changes (e.g. disinhibition, apathy) and neuropsychiatric symptoms (e.g. depression, psychosis) across a variety of dementia-related diseases  2. Review non-pharmacologic approaches for the management of behavioral and neuropsychiatric disturbances in dementia  3. Review pharmacologic strategies for managing neuropsychiatric symptoms in dementia |
|  |  |  | **Care Partner Resilience and Coping**  1. Understanding social and family (genogram) structure and dynamics: relational autonomy, family conflicts, stigma and social isolation.  2. Assess and address caregiver needs, burden,and burnout | **Supporting Care partners of People Living with Dementia**  1. Discuss common issues affecting care partners of people living with dementia  2. Describe the process of bereavement and grief (including anticipatory grief) among families and care partners of people living with dementia  3. Provide an overview of approaches for promoting care partner resilience in the context of neurological illness |
|  |  |  |  | End-of-Life Care in Advanced Dementia  1. Describe the palliative care needs of people living with advanced and end-stage dementia.  2. Examine issues related to dysphagia including artificial nutrition and hydration and pleasure feeding.  3. Explore the role of hospice care in end-of-life for patients with advanced dementia, including challenges to timely hospice referral |
| 9 | 49/M, classic motor neuron disease | **Motor Neuron, Nerve & Muscle Diseases**  1. Differentiate the various levels of lower motor neuron pathology with reference to the neuraxis chart  2. Explain motor neuron disease diagnosis and progression  3. Classify neuropathies and recognize diabetic, post-chemotherapy, inherited and inflammatory demyelinating categories  4. Classify myopathies and distinguish acute (reversible) metabolic, dystrophic and inflammatory conditions | **Respiratory symptom management**  1. Describe the pathophysiology of dyspnea, and other respiratory symptoms in persons living with neurological illness  2. Describe Breathing Thinking Functioning model of Dyspnea  3. Develop integrated care pathways for management of respiratory symptoms in patients with neurological illnesses | **Symptom management motor neuron disease Part I**  1. Identify the most common symptoms that people living with ALS experience, and which of these risk being underrecognized.  2. Describe common causes of pain in ALS  3. Propose a management plan for treating pain, dyspnea, spasticity, cramps, and fatigue using both nonpharmacologic and pharmacologic methods |
|  |  |  |  | **Symptom management motor neuron disease Part II**  1. Propose a management plan for insomnia, sialorrhea, cognitive changes, behavioral issues, depression, and anxiety using both nonpharmacologic and pharmacologic methods.  2. Identify sources of existential distress and care partner burden in the context of ALS |
|  |  |  |  | **Decision-Points and End of life care in motor neuron disease**  1. Discuss the American Academy of Neurology algorithms for respiratory and feeding management  2. Identify prognostic indicators relevant to hospice referral for patients with ALS  3. Identify the role of non-traditional means of symptom management at end of life, including non-invasive ventilation, and how this may differ from the hospice approach for other illnesses  4. Address the challenges and considerations involved in de-escalation off of the ventilator for awake ALS patients |
|  |  |  |  | **Requests for Assisted Death**  1. Define requests for hastened death, Medical Aid In Dying and Voluntary Active Euthanasia.  2. Identify some root causes of suffering that prompt requests.  3. Review the current legal status of requests for hastened death in various countries.  4. Apply a 6-step protocol for responding to requests |
| 10 | 20/M, Duchenne Muscular Dystrophy | **Childhood neurological illnesses: Transitions to Adulthood and Palliative Care**  1. Understand common issues of transition from pediatric to adult neurology.  2. Review the basic features of the following conditions in adolescence: cerebral palsy, epileptic encephalopathies, muscular dystrophies, spinal muscular atrophies, leukodystrophies and selected neurometabolic conditions (organic acidemias, mitochondrial dis, NCL). | **Spiritual Dimensions of Care**  1. Define spirituality and demonstrate the reflective capacity to understand its importance in everyone’s life  2. Assess and understand the patients/families/caregivers spiritual, existential and religious needs while recognizing and respecting boundaries  3. Integrate spiritual needs in the care plan and document provision of spiritual care. | **Management of dysarthria and dysphagia in neurological disease**  1.List management strategies for dysarthria and dysphagia in people living with neurological disease  2. Alternative and augmentative modes of communication  3. Summarize the important talking points in helping patients with neurological disease (ALS, dementia, PD) decide whether or not they should have a gastrostomy tube placed |
|  |  |  |  | **Rehabilitation in Neuromuscular Diseases**  1. Describe the role of rehabilitation medicine throughout the clinical phases of progressive neuromuscular illness (early, progressive, advanced/end-stage)  2. Compare and contrast the medical and social models of disability  3. Appreciate the potential advantages of integrated palliative care and rehabilitation medicine |
|  |  |  |  | **Setting up an interdisciplinary/multidisciplinary clinic for neurological disorders**  1. Compare the terms multidisciplinary and interdisciplinary  2. Appreciate the benefits and challenges of inter/ multidisciplinary care as opposed to physician--only neurology clinics  3. Identify key components of inter/multidisciplinary clinics necessary to provide high quality care for people living with serious neurological illnesses |
| 11 | 21/F. Multidrug resistant State III Tubercular meningo-arachnoiditis | **Infectious and Inflammatory Diseases**  1. Identify components of the meningitis syndrome; distinguish acute and chronic meningitis and list steps in treatment of acute meningitis  2. Recognize the encephalitis syndrome and identify acute viral and autoimmune encephalitides  3. Itemize inflammatory-demyelinating CNS diseases | **Hospice: Transitions and Organization**  1. Identify the components of hospice care and service gaps in the provision of end of life care, especially for patients with neurological illness  2. Discuss the roles and responsibilities of the hospice team.  3. Discuss triggers for transfer of patients to a Hospice. | **Neuropathic pain: Management Principles**  1. Examine the currently available pharmacological and non-pharmacological options for the treatment of neuropathic pain, including advanced techniques (methadone, ketamine, cannabis, interventional procedures)  2. Identify appropriate uses for opiates in the setting of neuropathic pain |
|  |  |  | **Grief and Bereavement support**  **1. Reco**gnize grief and bereavement in persons living with neurological illness and their families/care partners  2. Distinguish normal grief/bereavement from depression; and persistent complex grief/bereavement disorders  3. Understand the influence of different sociocultural practices on grief and bereavement | **Delirium in neurological disease**  1. Evaluate the risk factors for delirium in patients with serious neurological disease.  2. Consider the specific challenges of managing delirium in this population, with attention to pharmacological considerations and available options.  3. Evaluate the literature and formulate a management plan for terminal delirium in patients with neurological disease. |
|  |  |  |  | **Bladder and urinary cath management in neurological disease**  1. Identify symptoms of urinary dysfunction in neurological disease and their consequences if left untreated  2. Outline a pharmacologic and non-pharmacologic approach to manage urinary symptoms  3. Describe “red flags” that should prompt evaluation for causes of urinary dysfunction unrelated to neurological disease. |
| 12 | 40/M, Megalencephalic leukodystrophy | **Approach to Episodic Loss of Consciousness and Headache** 1. Distinguish various causes of transient loss of consciousness  2. Identify different seizure types and define approach to management  3. Recognize common primary and secondary headaches and list a diagnostic approach to secondary headaches | **Palliative Sedation**  1. Define palliative sedation and the principle of "double effect"  2. Identify triggers for palliative sedation  3. Demonstrate the process and documentation for palliative sedation in a patient with advanced neurological illness | **Clinician burnout, resiliency, and self-care**  1. Recognize signs and symptoms of burnout  2. Understand the benefits of self-care, and potential barriers to practicing self-care  3. Learn practices to build and maintain resilience |
|  |  |  |  | **Seizure management at the end of life**  1. Describe common clinical presentations of seizures in advanced illness  2. Implement strategies for appropriate use of maintenance antiepileptics in end stage illness, including when oral administration is not possible  3. Develop a framework for abortive medication management of acute seizure activity at end of life 4. Identify strategies for mitigating caregiver distress around acute seizure at home |
